# Ictal kissing with subdural EEG recording^☆^

**DOI:** 10.1016/j.ebcr.2013.05.001

**Published:** 2013-06-15

**Authors:** Abdulaziz Alsemari, Faisal Alotaibi, Salah Baz

**Affiliations:** Department of Neurosciences, King Faisal Specialist Hospital and Research Centre, Riyadh, Saudi Arabia

**Keywords:** Ictal kissing, Nondominant temporal lobe, Subdural EEG

## Abstract

**Purpose:**

Ictal kissing has been described in the literature. Five cases were reported and associated with temporal lobe epilepsy lateralizing to the nondominant hemisphere.

**Methods:**

A case of ictal kissing was identified. The aim was to demonstrate the clinical, clinical and electrophysiological features (as recorded by subdural electrodes). The surgical procedure, histopathology, and imaging data were reviewed and correlated with the literature.

**Results:**

A 29-year-old right-handed female, who presented with ictal right hand left arm dystonic posturing, and lip smacking, was studied. The automatism was usually followed by prolonged emotional gestures and by hugging and kissing her relative and/or attendant nurse. Magnetic resonance imaging of the brain showed right small cortical and subcortical lesions of the right inferior frontal lobe with gliosis but without mass effect and normal-sized hippocampi. The PET scan showed hypometabolism of the right temporal lobe. Neuropsychological evaluation showed deficit in her nonverbal memory. The subdural electrodes showed high amplitude spikes over right mesial temporal lobe strips. The offsite of the ictal discharges was usually at the right frontal strips. Right standard temporal lobectomy with amygdalohippocampectomy and right inferior frontal lesionectomy were performed. The patient continued to be seizure-free for one year postoperatively.

**Conclusion:**

Our case report supports with subdural EEG recording the findings of the few reported cases of ictal kissing behavior lateralized to the nondominant hemisphere. However, the affectionate kissing behavior was associated with spread of the epileptic discharges to the right frontal lobe.

## 1 Introduction

Automatisms with emotional behavior during seizures have been well described, including sudden changes in facial expression seen with chewing, manual automatisms, uncontrollable laughter, and ictal crying [1], [2], [3]. Ictal kissing is a rare phenomenon and has been described in previous patients [4], [5], [6]. The clinical significance and neurophysiological mechanisms of ictal kissing remain poorly understood. We describe a patient with ictal kissing and subdural EEG recording.

## 2 Clinical report

**Fig. 1:**
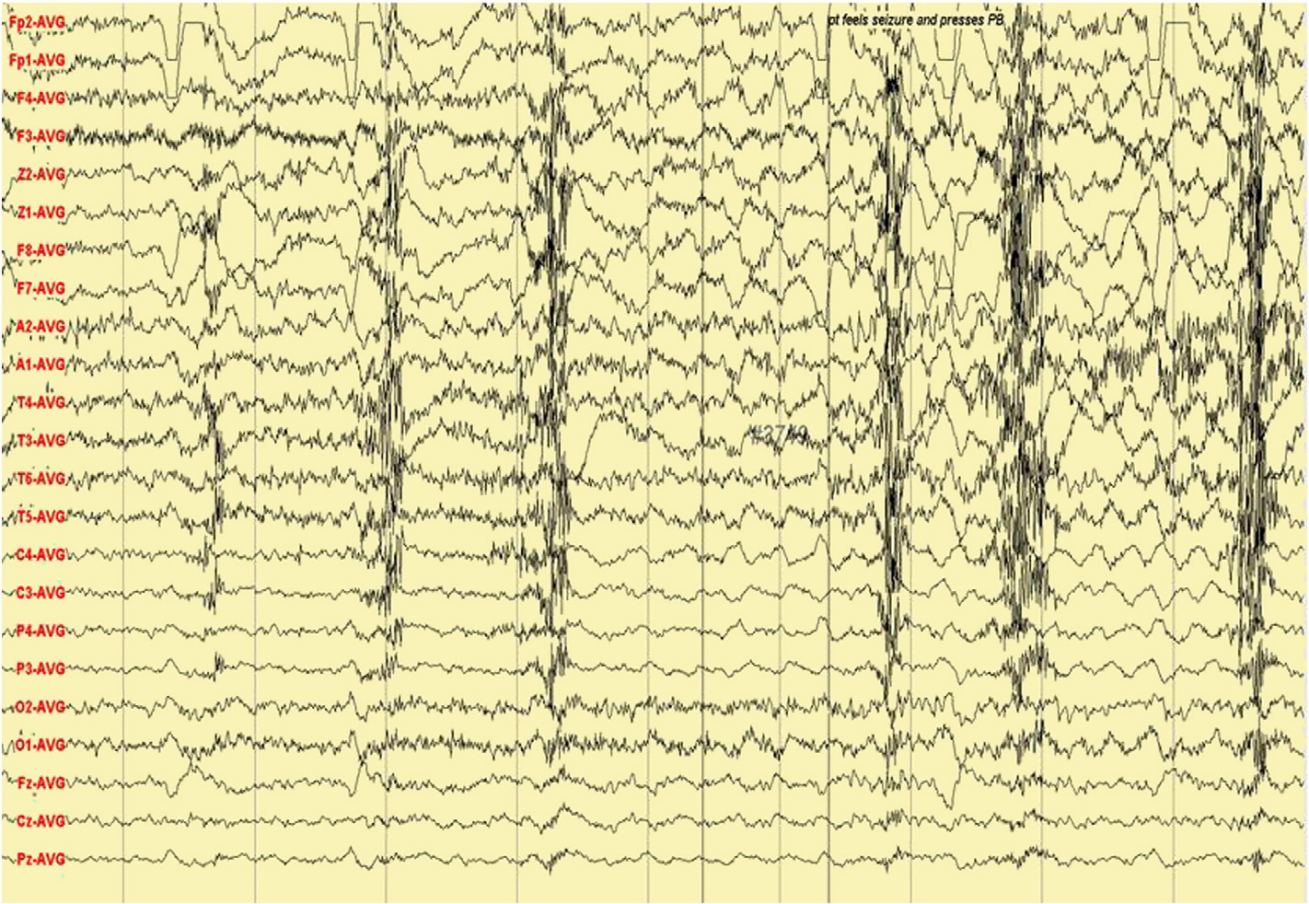
Surface ictal EEG onset shows rhythmic slow waves over the right frontotemporal areas.

A 29-year-old right-handed female, who has a history of epilepsy for the last 10 years, was studied. The patient has no history of febrile convulsions. She has a history of an aura of fear followed by automatism consisting of right hand left upper limb dystonic posturing, and lip smacking. The automatism was usually followed by prolonged emotional gestures, hugging and kissing her relative and/or attendant nurse. The patient's seizure frequency varied from 1 to 2 per month. Magnetic resonance imaging of the brain showed small cortical and subcortical lesions of the right inferior frontal lobe without mass effect or enhancement. The lesion was located directly anterior of the insular gyrus and inferior to the inferior frontal gyrus (Fig. 7). The hippocampi looked symmetrical (Fig. 8). The PET scan showed hypometabolism of the right temporal lobe (Fig. 9). Neuropsychological evaluation showed deficit in her nonverbal memory. The surface EEG showed right frontotemporal rhythmic slow wave activity (Fig. 1). Because of the uncertainty of the surface ictal EEG onset, the right inferior frontal lesion, and the normal-sized and symmetrical hippocampi, subdural EEG recording strips were done, covering the right temporal lobe and the right inferior frontal prelesional regions.

**Fig. 2:**
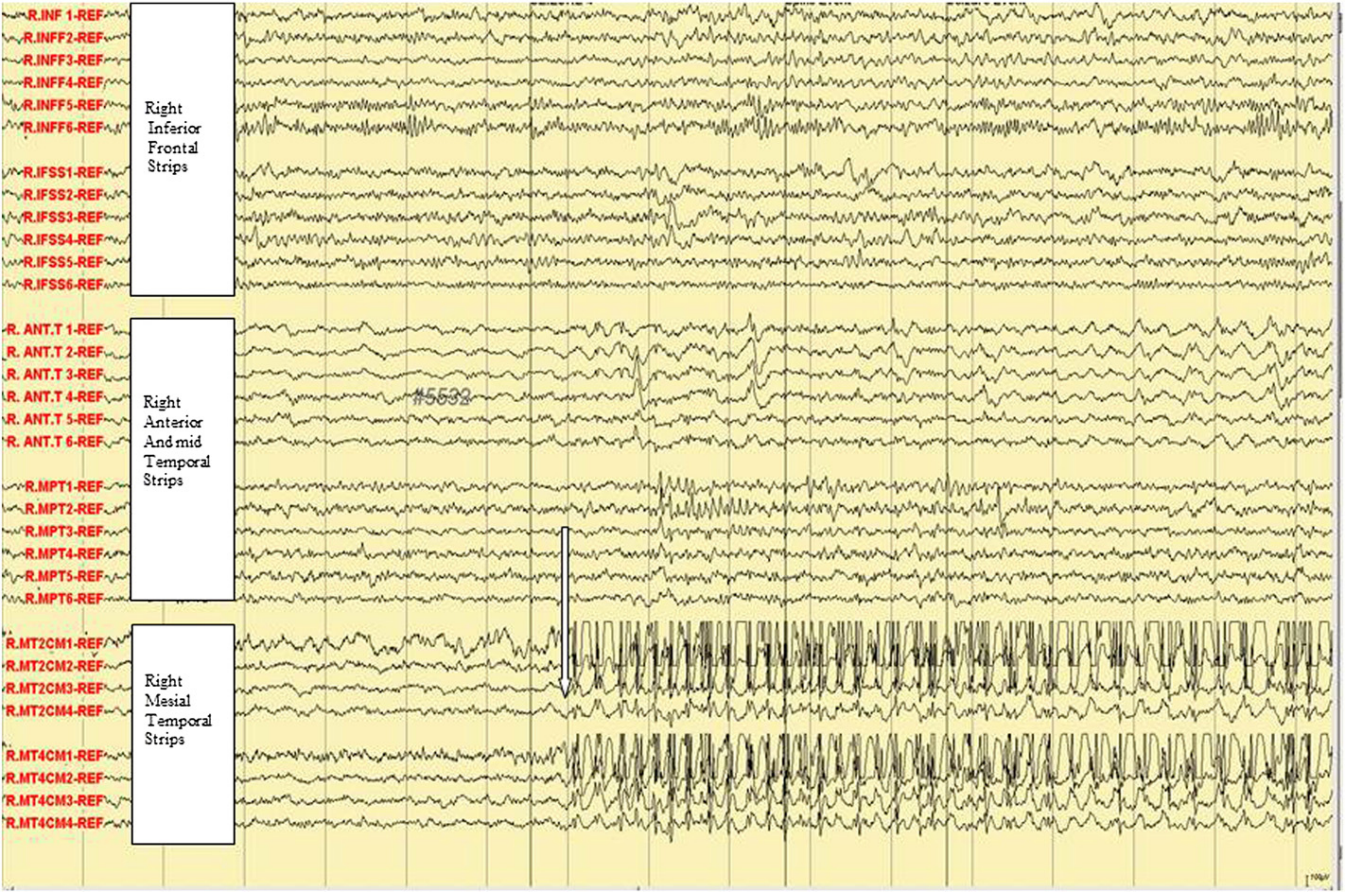
Ictal subdural EEG onset: aura of fear and palpitation.

**Fig. 3:**
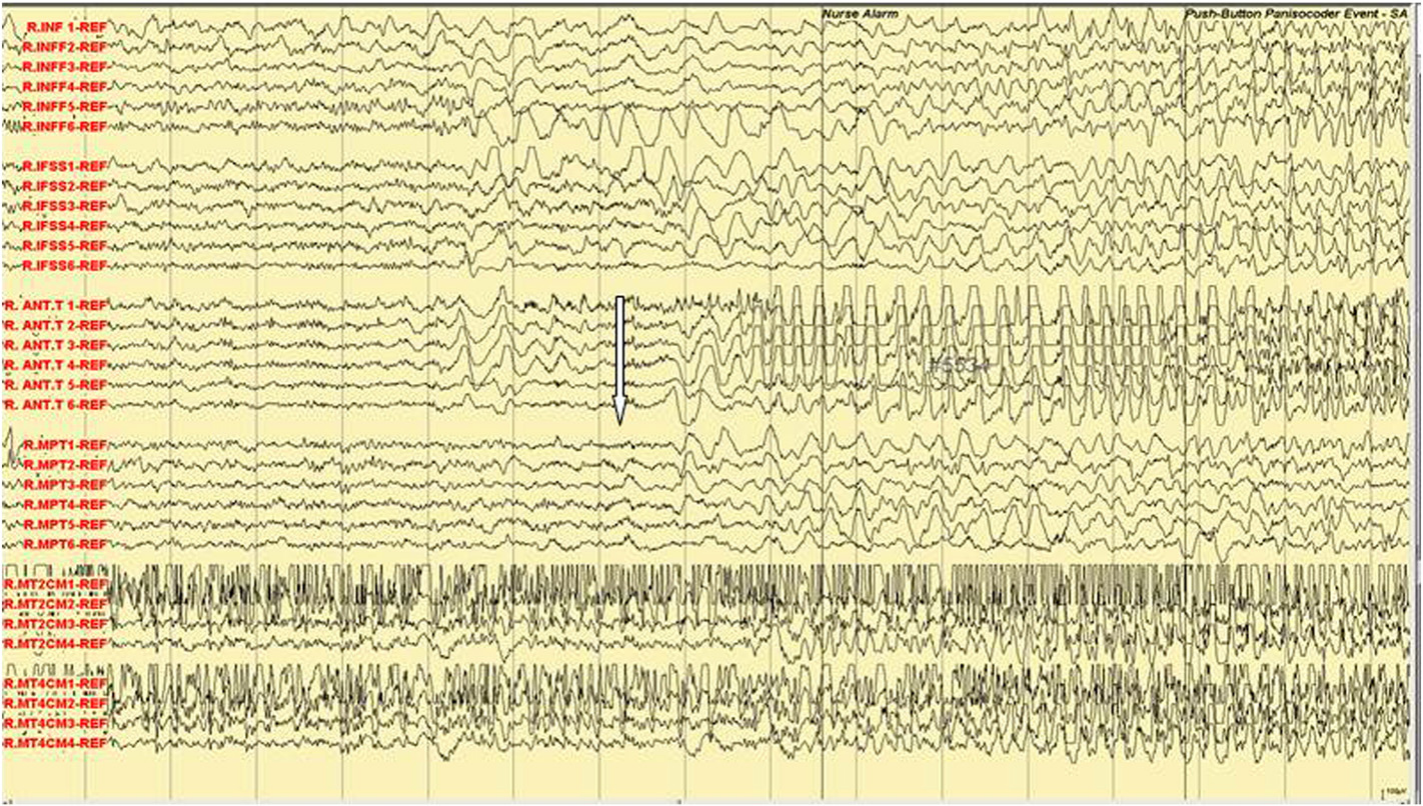
Ictal subdural EEG: manual automatism and lip smacking.

**Fig. 4:**
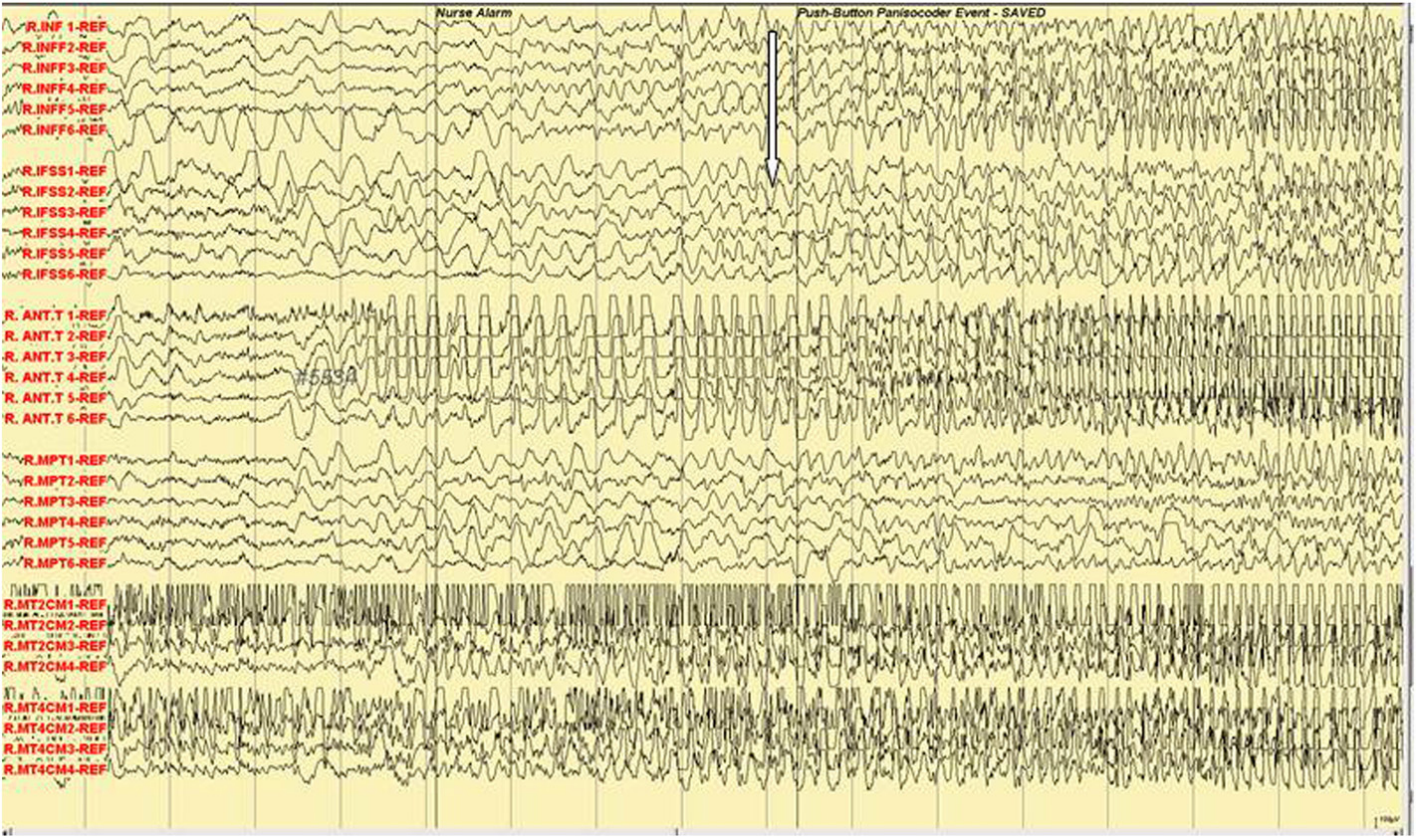
Ictal subdural EEG: hugging and kissing and left arm dystonic posturing.

**Fig. 5:**
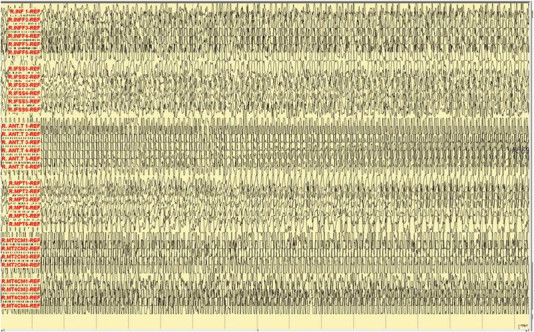
Ictal subdural EEG: more marked kissing behavior.

**Fig. 6:**
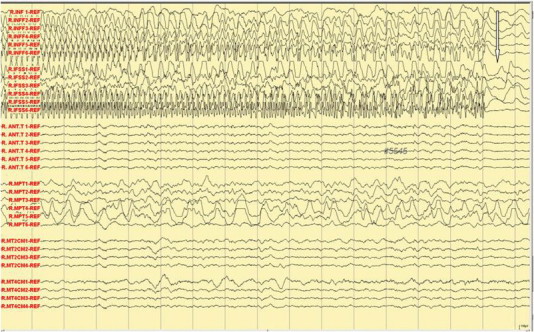
Ictal subdural EEG: end of the kissing behavior, no postictal confusion.

**Fig. 7:**
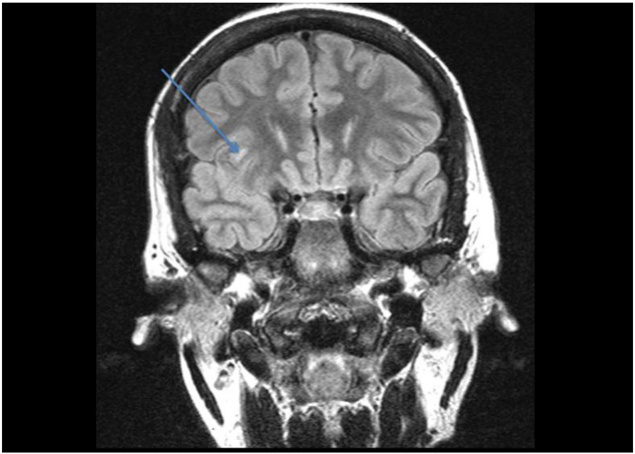
Brain MRI shows small cortical and subcortical lesions at the right inferior frontal lobe.

**Fig. 8:**
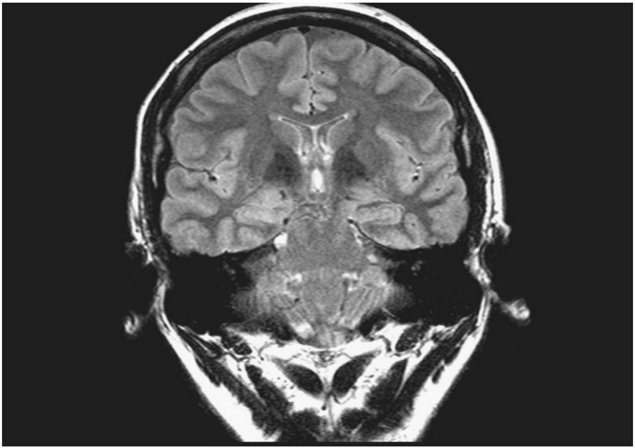
Brain MRI shows symmetrical normal-sized hippocampi.

**Fig. 9:**
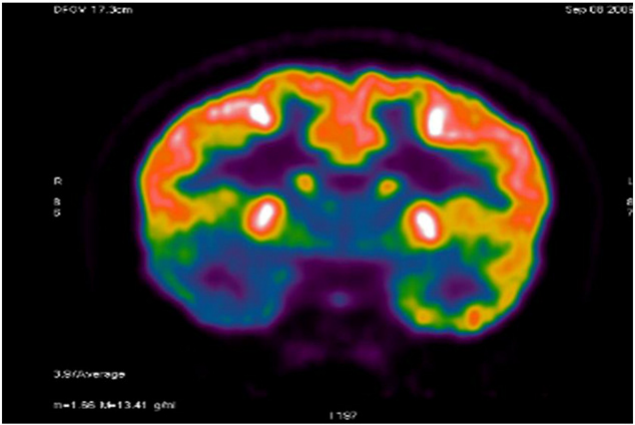
The PET study demonstrates decreased FDG metabolism involving the right temporal lobe.

## 3 Invasive video-EEG recording

In the Epilepsy Unit, 5 push button events were recorded. The subdural electrodes showed clinical correlation of fear and palpitation with an onset of high amplitude spikes over right mesial temporal lobe strips (R-MT2, 4) (see arrow, Fig. 2). The manual automatism and lip smacking started at the appearance of the epileptic discharges at the anterior temporal strip (see arrow, Fig. 3). The emotional hugging and kissing behavior was correlated with the spread of the epileptic discharges in the frontal lobe (see arrow, Fig. 4) and (Fig. 5). The end of the kissing behavior was coupled to the offsite of the ictal discharge at the right frontal strips (see arrow, Fig. 6).

## 4 Epilepsy surgery

Right standard temporal lobectomy with amygdalohippocampectomy and lesionectomy of the right inferior frontal lobe under neuronavigation guidance was performed. The patient continued to be seizure-free for one year. Histopathology of the hippocampus and the inferior frontal lesion was compatible with gliosis.

## 5 Discussion

Fear, anxiety, and emotional distress are among the most frequently reported behaviors in epilepsy. However, varieties of ictal automatisms with other emotional elements also have been described [7], [8]. Religious experiences occurring in patients with temporal lobe epilepsy are also described [9]. In addition, the association of ictal kissing with religious speech in a patient with right temporal lobe epilepsy has been well characterized [5].

The kissing and hugging behavior is an interesting feature of temporal lobe epilepsy and has been described in the literature [4], [5], [6]. Five cases were reported and associated with TLE lateralizing to the nondominant hemisphere. Three of them were associated with mesiotemporal sclerosis, one with a benign tumor, and one with a normal MRI. Our case is considered another example of ictal kissing with normal and symmetrical size hippocampi in brain MRI. The histopathology of the right hippocampus showed mild gliosis, and there was a gliotic lesion in the inferior frontal lobe, just anterior to the insula; however, it was not related to the ictal EEG onset. Three out of the five reported cases were operated, and the histopathologies were hippocampus sclerosis, cortical dysplasia, and low grade astrocytoma (Table 1).

**Table 1:** Clinical data of the reported cases.

	Case 1 Rashid et al. [6]	Case 2 Rashid et al. [6]	Case 3 Rashid et al. [6]	Case 4 Ozkara et al. [5]	Case 5 Mikati et al. [4]	Case 6 Alsemari et al. 2013
Age (years)	39	46	48	25	24	29
Imaging findings	MRI: no abnormality	MRI: R mesial temporal sclerosis	MRI: R mesial temporal sclerosis	MRI: R mesial sclerosis,	MRI: R mesial temporal lesion	MRI: small cortical and subcortical lesions at the right inferior frontal lobe without mass effect or enhancement; the hippocampi looked symmetrical
Histopathology	Cortical dysplasia type IIa	No surgery	No surgery	Hippocampal sclerosis	Low grade astrocytoma	Mild gliosis
Interictal EEG	SW, R temporal	SW, R temporal	SW, R > L temporal	SPK, R > L temporal	Unknown	SW, R temporal
Ictal EEG	Rhythmic theta activity maximum at F8-T8 and SP2, evolving into a high amplitude 7-Hz rhythm maximum over the R temporal region	Rhythmic theta activity beginning in the R temporal region, spreading to the frontal lobes (R > L) and evolving into polymorphic slowing	Theta activity in the R frontotemporal region evolving into rhythmic generalized slowing	Semi rhythmic theta activity over the right frontotemporal region, evolving to bifrontotemporal slowing	Rhythmic theta activity maximum F8 and SP2, in some seizures evolving to bilateral frontotemporal slowing with right-sided predominance	High amplitude spike discharges over right mesial temporal lobe strips spreading to the frontal lobe strips

The kissing ictal behavior was similar to typical behaviors seen with complex partial seizures, such as chewing, lip smacking, fumbling, tapping, rubbing, or other semipurposeful, repetitive movements. Our subdural EEG recording showed that the ictal epileptic discharge onset was at the right temporal lobe, and it was correlated well with the common manual and mouth automatism. Nevertheless, the spread of epileptic discharges in the frontal lobe was associated with the kissing and the affectionate behavior.

Epileptic seizures may cause dysfunction not only through excitation but also by inhibition of neuronal activity that extends beyond the area directly involved in the electrographic seizure activity, leading to behavioral dysfunction [6]. Inhibition of neuronal activity extends sometimes into the postictal period and can cause ongoing release of behavior control and, not uncommonly, postictal automatisms.

The affectionate behavior is a rare clinical manifestation in epilepsy; however, it is of scientific value in defining the cerebral circuitry of emotion. The feeling of affection for attachment and the underlying basis of human behavior have become important research themes in neuroscience. After the introduction of functional magnetic resonance imaging (fMRI), neuroscientists have demonstrated increased interest in the neurobiology and neurochemistry of emotions, including love and affection [10]. Epilepsy with kissing and sexual behavior is considered exaggerated behavioral phenomena and may assist in facilitating the discovery of the outlines of the physiology of romance.

Few fMRI studies elucidating neural correlates of romantic and affection have been published [11], [12], [13], [14], [15], [16], [17]. These studies are difficult to compare because they suffer from selection bias. However, some general conclusions can be drawn from these neuroimaging studies. The brain areas that show activation in romantic love were the medial insula, anterior cingulate cortex, hippocampus, striatum, nucleus accumbens, and hypothalamus.

In conclusion, our findings support the idea that the ictal kissing behavior is a phenomenon of nondominant temporal lobe epilepsy. However, it is possibly linked to a circuit involving regions beyond the temporal lobe, such as the medial insula, anterior cingulate cortex, medial orbitofrontal, and hippocampus in the nondominant hemisphere.
